# Characterizing temporal stability of supercontinuum generation in higher-order modes supported by liquid-core fibers

**DOI:** 10.1038/s41598-024-75249-9

**Published:** 2024-10-13

**Authors:** Johannes Hofmann, Ramona Scheibinger, Markus A. Schmidt

**Affiliations:** 1 Leibniz Institute of Photonic Technology, Albert-Einstein-Str. 9, 07745 Jena, Germany; 2 Otto Schott Institute of Materials Research (OSIM), Friedrich-Schiller-University Jena, Fraunhoferstr. 6, 07743 Jena, Germany

**Keywords:** Nonlinear optics, Solitons, Supercontinuum generation

## Abstract

The generation of tailored supercontinua is essential for studying ultrafast light-matter interactions and for a variety of practical applications requiring broadband light. Liquid-core fibers (LCFs) have emerged as an innovative nonlinear photonic platform, demonstrating high efficiency in nonlinear frequency conversion. In this study, we showcase that LCFs provide a stable platform for ultrafast supercontinuum generation in a selected higher-order vector mode at $1.55\;\upmu \hbox {m}$. Specifically, we demonstrate soliton fission and double-dispersive wave generation using a radially polarized mode in a $\hbox {CS}_{2}$-silica liquid-core fiber. The experiments were performed in a temperature-controlled laboratory, showing excellent stability with no evidence of fiber degradation, material degradation, or drift-induced changes in mode excitation over extended periods under standard environmental conditions. Our results confirm that liquid-core fibers are a reliable platform for nonlinear photonics, suitable for applications such as computationally tailored supercontinuum generation, single pulse spxectroscopy, and tailored light sources, all of which rely on consistent and stable nonlinear frequency conversion.

## Introduction

Supercontinuum generation (SCG) using nonlinear optical effects is an important and well-known mechanism for the frequency broadening of optical pulses^1^. It shows interesting physical effects (e.g. soliton fission^2^, soliton self-frequency shift^3^, nonlinear mode coupling^4,5^) and is of importance in various fields such as spectroscopy^6,7^, nonlinear imaging^8^, optical metrology^9–11^, telecommunications^12,13^ and non-invasive medical diagnosis^14,15^.

Due to the long light-matter interaction length and the possibility to tune the dispersive properties very precisely, this mechanism is particularly effective in optical fibers. Broadband SCG has been demonstrated, besides silica fibers, in soft-glass fibers^16–18^, gas-filled fibers^19,20^ and liquid-filled fibers^21^. Well-established solid glass (silica) fibers suffer from limitations such as low nonlinearity and limited transmission bandwidth^22–24^. Liquid-core fibers (LCFs) with step-index profiles represent a new type of optical fibers with unique properties such as wide transmission windows in the mid-infrared^24,25^, high nonlinearity^26–29^ and non-instantaneous response^26–28^. Straightforward realization by capillary filling and dispersion adjustment by liquid mixing^30,31^ or temperature modifications due to a high thermo-optic coefficient^32^ are further benefits of the new fiber type. The most commonly used and well-studied liquid in LCFs is $\hbox {CS}_{2}$ in combination with silica fiber-type capillaries, which exhibit low modal coupling and interference due to the high refractive index contrast between core and cladding^33^. More information about the current status of (here used) LCFs can be found here^21^.

However, if using the fundamental mode, this type of fiber neither exhibits suitable dispersion properties in order to observe e.g., soliton processes at telecom wavelength $1.55\;\upmu \hbox {m}$ nor supports strong dispersion tuning properties. Therefore, SCG in higher-order modes (HOMs) has recently attracted substantial attention and represents a new field of research in the area of nonlinear photonics, allowing for the observation of new physical effects (e.g., thermodynamics in nonlinear systems^34^, intermodal dispersive wave generation^35^) or the development of complex and tunable dispersion landscapes. It has been shown that using HOMs in $\hbox {CS}_{2}$-based LCFs leads to a specific dispersion landscape, which is particularly advantageous for SCG and difficult to achieve with solid-core step-index silica glass fibers. For example, effective power transfer from a soliton to two dispersive waves (DWs) has been measured in $\hbox {CS}_{2}$-based LCFs^36^. A special feature of $\hbox {CS}_{2}$ is the strong thermo-optic coefficient^32^, which is about 10-times higher compared to fused silica^37^ and allows a strong modulation of the refractive index. Thus, the dispersion of HOMs changes drastically with temperature compared to the fundamental mode. This opens up the possibility of adapting SCG flexibly and dynamically to specific requirements by external control^38^, which is unique and a strong benefit compared to other dispersion tailoring methods such as using complex fiber structures, tapered fibers etc.

In order to provide reproducible and comparable spectral outputs, stable and reliable SCG, including the incorporation of external influences (e.g., temperature changes), is crucial. This is particularly important in an application perspective when the system is manipulated by optimization algorithms, which are time-consuming and need to ensure comparability. Typically, stability is more critical for HOMs as the different core/cladding power distribution causes a greater dependence on external influences. So far, the stability has only been demonstrated in the context of LCFs in the fundamental ($\hbox {HE}_{11}$-) mode at a pump wavelength of $1.95\;\upmu \hbox {m}$^39^. It is important to note that all studies on HOMs and almost all works on LCFs in general operate at $1.55\;\upmu \hbox {m}$, which defines a clear motivation to investigate the stability of SCGs in LCFs for HOM excitation.

**Fig. 1:**
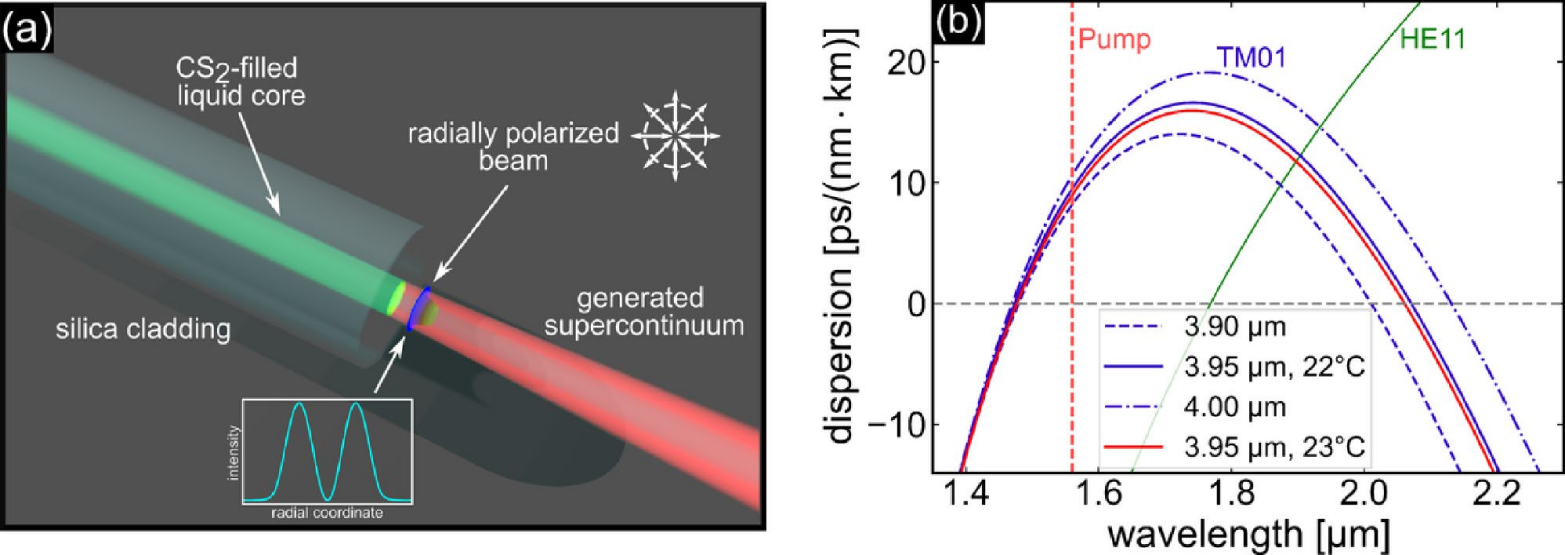
(**a**) Illustration of the concept of higher-order mode supercontinuum generation in liquid-core fibers. The green cylinder refers to the $\hbox {CS}_{2}$-filled core, while the red beam indicates the broadband output light that has a doughnut shape intensity distribution. The sketch with the double-headed arrows indicates the radial polarization states of the generated supercontinuum. The inset shows the doughnut-shaped intensity distribution of the vector mode considered in this work. (**b**) Group velocity dispersion of a selected HOM ($\hbox {TM}_{01}$-mode) of a $\hbox {CS}_{2}$/silica LCF for three different core diameters and two temperatures (dashed: $3.90\;\upmu \hbox {m}$, solid: $3.95\;\upmu \hbox {m}$, dotted-dashed: $4.00\;\upmu \hbox {m}$, blue: $T = 22\;^{\circ }\hbox {C}$, red: $T = 23\;^{\circ }\hbox {C}$). The green curve refers to the dispersion of the fundamental mode ($\hbox {HE}_{11}$-mode, $3.95\;\upmu \hbox {m}$).

In this work, we show that LCFs provide a stable and reliable platform for ultrafast SCG in a selected HOM. Specifically, we demonstrate soliton fission and double DW formation using a higher-order vector mode ($\hbox {TM}_{01}$-mode) in a $\hbox {CS}_{2}$-silica LCF in a temperature-stabilized laboratory (Fig. 1a). No evidence of fiber or material degradation or drift-induced changes in mode excitation was observed over 24 hours under conventional environmental conditions.

## Concept

The spectral broadening approach used here is based on the fission of higher-order solitons in the anomalous dispersion (AD) region and the simultaneous generation of DWs in the normal dispersion (ND) domain with high efficiencies^19,20,40,41^. Here, the spectral position of the DWs is described by a phase matching condition^1,42^. The key to SCG is the spectral distribution of the group velocity dispersion (GVD) of each mode, with the zero crossings, the so-called zero dispersion wavelength (ZDW), being significant benchmark indicators. The use of HOMs in combination with LCFs allows the formation of a complex dispersion landscape with a defined region of AD at $1.55\upmu \hbox {m}$, which is bounded by two ZDWs (blue curves in Fig. 1b)^36^. The dispersion of simple step-index type fibers can be calculated analytically for the respective mode^43^ using refractive index data of $\hbox {CS}_{2}$^33^ and $\hbox {SiO}_{2}$^44^. Thus, the soliton fission is spectrally defined and the spectral position of the DWs can be controlled by influencing the dispersion, i.e., the ZDWs. As shown in a previous work, HOMs show a strong temperature dependence that can be exploited to tune the output spectra^38^. It is important to note that the fundamental ($\hbox {HE}_{11}$-) mode shows a purely monotonically increasing GVD (green curve in Fig. 1b) and a significantly reduced influence on external parameters (e.g., core diameter and temperature), making it uninteresting for modulation experiments.

## Methods

The LCFs used here consist of fiber-type silica capillaries with a central micrometer-size hole, which are filled with $\hbox {CS}_{2}$ by capillary force. Note that the Washburn equation^45^, which considers fiber length and diameter, viscosity and contact angle, can be used to estimate the filling time. The empty capillary was fixed in custom-made optofluidic holders, which contain a liquid reservoir and allow optical access through integrated sapphire windows. In the present experiments, LCFs (core diameter ${3.95}\;\upmu \hbox {m}$) of about $15\;\hbox {cm}$ length were realized by filling the fiber with $\hbox {CS}_{2}$ from one of the reservoirs.

**Fig. 2:**
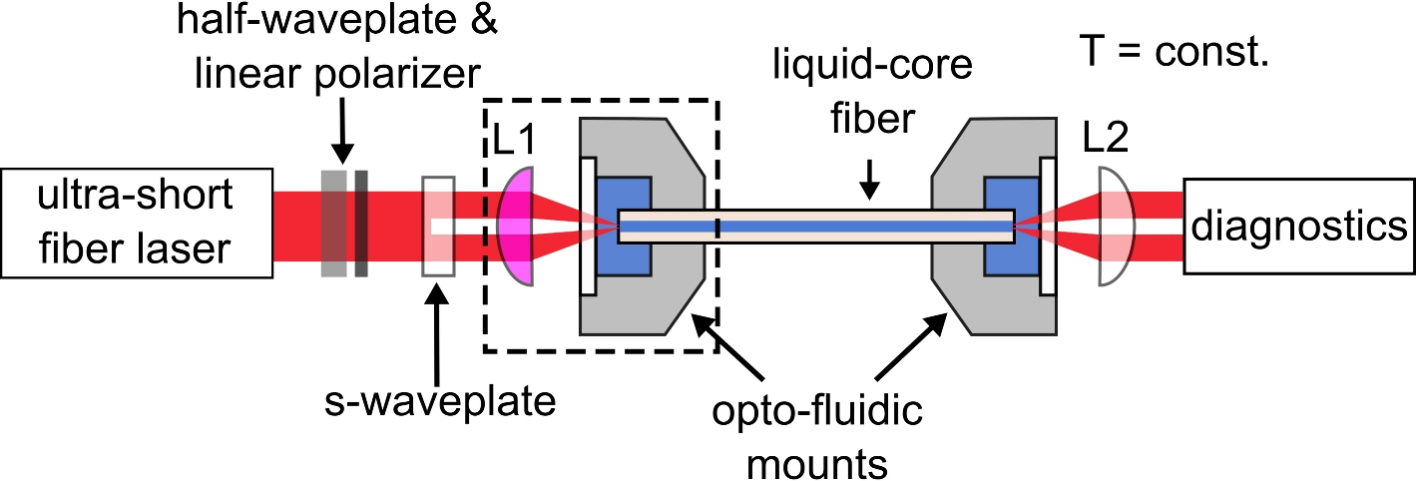
Optical setup for the characterization of the stability of HOM supercontinuum generation in LCFs. The setup includes an ultrafast laser, polarization optics, mirrors (not shown), an s-waveplate for polarization conversion, aspheric lenses mounted on xyz-stages (L), the sample including opto-fluidic mounts and diagnostics. The critical incoupling optomechanical components are depicted by a dashed box, in particular the lens L1 mounted on xyz-stage is highlighted in pink. The ambient temperature was kept constant between ${22.1}\;^{\circ }\hbox {C}$ and $22.3\;^{\circ }\hbox {C}$.

Pulses from an Er-doped fiber laser (central wavelength $\lambda = 1.56\;\upmu \hbox {m}$, pulse width ${30}\;\hbox {fs}$ (FWHM), repetition rate ${80}\;\hbox {MHz}$, FemtoFiber pro IRS-II) were used for the nonlinear experiments to demonstrate the long-term stability of the SCG process (Fig. 2). To transform the linear polarization of the input light into a desired vector polarization corresponding to the polarization of the mode to be excited, an s-waveplate was inserted into the beam path^46^. Depending on the orientation of the s-waveplate relative to the input polarization, the resulting vector polarization is either radial or azimuthal. In this work, the $\hbox {TM}_{01}$-mode (radial polarization) is excited in a fiber which supports $M \approx V^2/2 = 13$ modes (including degenerated modes) at the pump wavelength. Note that modal overlap calculations of a similar LCF system have confirmed highly selective mode excitation, where a radial polarized input beam leads to power fraction of $99\%$ for the $\hbox {TM}_{01}$-mode, while only $1\%$ is transferred into the $\hbox {HE}_{21}$-mode^36^. Additionally, mode images do not indicate any contribution of the $\hbox {HE}_{11}$-mode. Note that the same concept is also valid for, e.g., the $\hbox {TE}_{01}$-mode (azimuthal polarization) and it is solely a matter of choice.

Input and output coupling is provided by aspheres (Thorlabs C230-C: $f = {4.5}\;\hbox {mm}$, $\text {NA} = {0.55}$ and C036-D: $f = {4.0}\;\hbox {mm}$, $\text {NA} = {0.56}$) mounted on high-precision xyz-stages. An average power of ${100}\;\hbox {mW}$ is coupled into the LCF, which corresponds to a peak power of ${41}\;\hbox {kW}$ assuming a Gaussian pulse shape. The coupled power fraction (coupling efficiency) into the desired fiber (here $\hbox {TM}_{01}$-mode) mode is estimated by measuring the power in front of the first lens and after the second lens (at the output side). Possible losses contributions that do not originate from the LCF like transmission and reflection losses in both lenses (using available data provided by Thorlabs), as well as reflection at the glass windows surfaces are included in order to correct the launched and extracted power values. No loss inside the fiber is assumed due to the high transparency of the liquid^47^. That approach leads to $31\%$ of power coupling into the $\hbox {TM}_{01}$-mode, resulting in a soliton number of $N_{\text {sol}} = 3$ at a GVD of ${10}\;\hbox {ps}/(\hbox {nm} \cdot \hbox {km})$^42^.

To obtain a full spectral characterization of the generated spectra, a reflective objective was used once in combination with a FTIR spectrometer to measure the full bandwidth without chromatic aberrations. For the stability measurements, the FTIR was replaced by an optical spectrum analyzer (OSA, Yokogawa AQ6375B ($1\;\upmu \hbox {m}$ to ${2.5}\;\upmu \hbox {m}$)) and the reflective lens was replaced by an asphere. Due to the chromatic aberrations of the lens, the coupling to the OSA is wavelength-dependent, and a long pass filter is required to suppress the higher diffraction orders in the OSA. For the spectral measurements, the system has been optimized on the long wavelength side of the spectrum (i.e., the long wavelength dispersive wave, DW), as this is expected to provide all relevant information for assessing stability. Three key factors can potentially affect the spectral output: (1) mechanical drift and incorrect mode excitation, (2) material damage, and (3) thermal dispersion changes. Mechanical drift and material damage would affect both the short and long wavelength sides similarly as any fiber degradation or mode excitation problems would be visible throughout the spectrum. Thermal dispersion changes, however, are more likely to affect the long wavelength side, as shown in Fig. 1b, where the dispersion shifts more significantly at longer wavelengths. For these reasons, analysis of the long wavelength side alone is sufficient to provide information about the stability of the system under study. Spectra were recorded every 10 min, and the laser was never turned off during experiments. An important aspect is to ensure a constant ambient temperature, which was kept stable between ${22.1}\;^{\circ }\hbox {C}$ and ${22.3}\;^{\circ }\hbox {C}$ for all experiments. On the one hand, this is important for SCG itself, since the HOM is highly temperature-dependent. On the other hand, the coupling takes place via mechanical components that slightly move with changing environmental conditions and thus influence the excitation of the highly sensitive HOM.

## Results and discussion

**Fig. 3:**
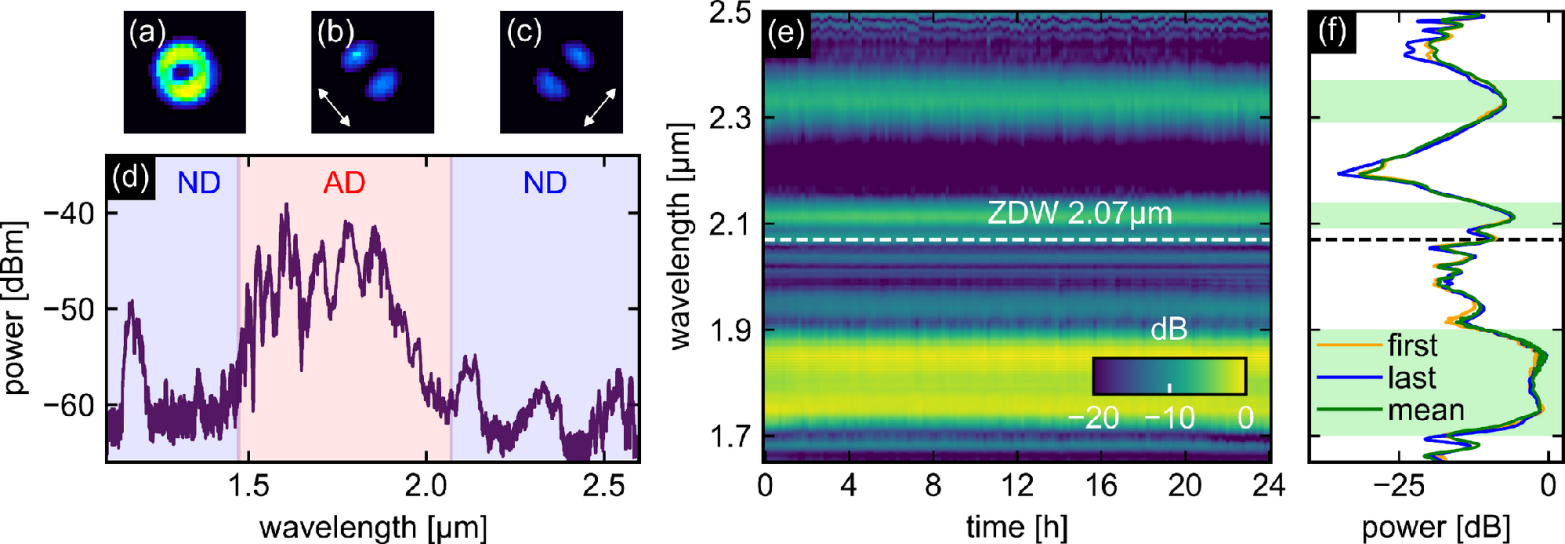
Results of the supercontinuum experiments demonstrating the stability of the generated spectra. The images (**a**–**c**) on the left show the mode patterns in the spectral domain ${0.9}\;\upmu \hbox {m}< \lambda < {1.7}\;\upmu \hbox {m}$. (**a**) shows the case where no polarizer is inserted in the beam path, while (**b**) and (**c**) show the distribution for different polarizer orientations (double-headed arrows). (**d**) Example of a measured output spectrum (details in the main text). The blue (red) background refers to ND (AD). (**e**) Stability-related HOM-supercontinuum measurement using the LCF and laser source described in the main text, showing the spectral distribution of the output light over a period of 24 h. (**f**) Spectral distribution of three selected output spectra (orange: first spectrum at the beginning of the measurement series, blue: last spectrum taken after 24 h, green: average spectrum obtained from all spectra considered in this work). The light green areas indicate the three spectral intervals that contain intense spectral features and thus have been considered in the statistical analysis.

To confirm that $\hbox {TM}_{01}$ mode used here allows for broadband SCG, the full bandwidth of the generated spectrum was measured at ${100}\;\hbox {mW}$ input power in the described LCF (Fig. 3d). The appearance of different spectral features corresponding to solitons and multiple DW (in the long- and short-wavelength ND regime) can be clearly seen. The corresponding mode image (Fig. 3a) shows a ring-shaped intensity distribution in the spectral region ${0.9}\;\upmu \hbox {m}< \lambda <{1.7}\;\upmu \hbox {m}$, which is clearly due to the excitation of a $\hbox {TM}_{01}$-mode. This is confirmed by the mode image obtained by placing a polarizer between the camera and the sample (Fig. 3b,c). For different orientations of the polarizer, two lobes can be seen that rotate with the polarizer axis.

In order to demonstrate the long-term stability of $\hbox {TM}_{01}$-mode SCG, multiple spectra were recorded over a period of 24 hours (Fig. 3e). All spectral features remained at the same spectral position and no change in the power values of the different features was observed. This effect is also evident when comparing the first spectrum taken at the beginning of the measurement series (orange line in Fig. 3f) with the last spectrum taken after 24 h (blue line in Fig. 3f), together with the average spectrum obtained from all measurements (green line in Fig. 3f). As shown (a similar comparison using a linear scale can be found in Supplementary Information Sec. S1), (1) the first and last spectra overlap significantly, and (2) the average spectrum closely matches the two individual spectra, confirming the stability of the system. To further quantify the stability, a statistical analysis of the SCG was performed. The analysis focused on the spectral intervals containing the highest power, as these are typically the most important (highlighted in light green in Fig. 3f, defined in Table 1). To summarize the temporal variations, the standard deviation was calculated for each relevant wavelength and then averaged over the spectral interval of interest. This approach yields the mean relative standard deviation (RSD) and provides a clear assessment of the fluctuations within the interval (details in Supplementary Information Sec. S2). The results (shown in Table 1) show very low mean RSD values, indicating minimal variation over time and confirming the overall stability of the system. It turned out that the stability of the temperature (${22.2}\;^{\circ }\hbox {C}\pm {0.1}\;^{\circ }\hbox {C}$), is crucial, as changes in the spectrum occur for larger temperature fluctuations. To explain this, two different influences must be considered: On the one hand, the dispersion landscape of the fiber changes with temperature, which can lead to a change in the output spectrum. This is not a problem for demonstrating the stability of the fiber against degradation discussed in this work, since a change in GVD would only shift the spectral components. However, for long-term measurements, changes in spectral characteristics must be carefully considered when quantifying comparability and reproducibility. By calculating the GVD for a temperature difference of $\Delta T = {1}\;\hbox {K}$, which is greater than the maximum variation in the laboratory, the resulting dispersion is almost unchanged (compare the overlap of the blue and red curves in Fig. 1b). Note that, for the selected fiber, the ZDWs shift by ${5}\;{\frac{\hbox{nm}}{\hbox{K}}}$ and ${12}\;{\frac{\hbox{nm}}{\hbox{K}}}$, respectively. Therefore, if the temperature changes by $\Delta T = {1}\;\hbox {K}$, no significant effect on the generated spectrum due to a changed dispersion is expected. Note that larger temperature changes are required to use temperature tuning for real-time control of the SCG. On the other hand, temperature induced spectral changes can be caused by a thermal drift of optomechanical components particularly at the input side, leading to altered coupling conditions and a different mode excitation, which is especially critical in the case of HOMs. This results in (1) a modified spectral output, which is critical in the context of comparability and reproducibility, and (2) possibly a complete suppression of HOM and purely fundamental $\hbox {HE}_{11}$ mode excitation, which is additionally relevant for the investigation of HOM damage thresholds.

**Table 1:** Results of the statistical analysis of the output power in the spectral intervals of highest power ($N_s = 134$).

	$\lambda _{\text {start}}$ [nm]	$\lambda _{\text {end}}$ [nm]	$\Delta \lambda$ [nm]	$N_{\lambda }$	$\overline{RSD}(\Delta \lambda )$
Interval 1	1700	1900	200	1001	0.088
Interval 2	2090	2140	50	251	0.052
Interval 3	2290	2370	80	401	0.086

Neither spectral changes nor a change of the ring-shaped mode profile were measured during the first 24 h, indicating a stable system regarding optomechanical components as well as no material damage or degradation. After more than 24 h of measurement, a gradual change in the measured spectral output was observed, which can be correlated with a change in ambient temperature. By slightly readjusting the input, the original spectrum could be restored, thus ruling out any possible damage to the sample. It should be noted that different samples were used in the same experimental configuration over longer periods of time (several months) and no irreversible changes were observed. Overall, it has been shown that HOMs in the LCF allow for stable SCG over long durations using the experimental setup. As described above, possible changes in the output spectra result from changes of the incoupling, which can in principle be eliminated by integrating the LCF into the fiber circuit in order to have an all-fiber system. Such integration has been recently achieved by one of the authors through a combination of liquid volume reduction by cooling, splicing and expansion^48^. This enables LCFs to be used in conventional fiber circuitry, opening up new applications in areas such as material research, life science and nonlinear optics. In general, LCFs offer several advantages over conventional all-glass fibers (a detailed review on LCF can be found in Ref.^21^). They offer reconfigurable and real-time dispersion tunability due to the high thermo-optic response of the liquid core^38^. Waveguide dispersion and nonlinearity can be precisely controlled by filling the core with a mixture of liquids^30^. LCFs are easily implemented by leveraging the capillary effect in simple fiber-like capillaries, requiring no additional post-processing^21^. Efficient axial dispersion management is achieved by partially collapsing the core prior to liquid filling^49^. LCFs also prevent stress-induced birefringence, which improves polarization performance^50^. In addition, LCFs enable tailored group velocity dispersion, ideal for soliton-based supercontinuum generation, including the creation of an anomalous dispersion region at $1.55\;\upmu \hbox {m}$ with adjustable zero dispersion wavelengths (ZDWs)^36^. They offer high infrared transmission due to the extended windows of inorganic solvents, surpassing fused silica^47^. The non-instantaneous response supports novel nonlinear phenomena such as hybrid solitons and more stable supercontinua^51^. In addition, their narrowband Raman response could enable efficient Raman shifters^52–54^.

## Conclusion

Stable and reliable generation of supercontinua is crucial for using this nonlinear frequency conversion scheme in the context of studying fundamental light-matter interaction and real-world applications. In this work, we show that liquid-core fibers provide a steady platform for the ultrafast generation of soliton-based supercontinua in a selected higher-order mode. Specifically, we demonstrate soliton fission and double dispersive wave generation by using a radially polarized vector mode in a $\hbox {CS}_{2}$-silica liquid-core fiber over the course of more than 1 day. Using a temperature-stabilized laboratory, we show that there is no evidence of fiber and material degradation, nor any influence of drift-induced changes of mode excitation over a measurement period of more than 24 h. Changes in the spectrum are exclusively due to changes in the coupling optics, which can be compensated by readjustment. This study clearly demonstrates that liquid-core fibers are a robust and stable platform for nonlinear photonics that can be used for the realization of tailored supercontinua through computer-assisted optimization, nonlinear experiments with reconfigurable dispersion to investigate new physical effects, single pulse spectroscopy, or tailored light sources.

## Supplementary Information

Supplementary Information.

## Data Availability

The datasets generated during and/or analysed during the current study are available from the corresponding author on reasonable request.
