# Supplementary material for: Characterizing temporal stability of supercontinuum generation in higher-order modes supported by liquid-core fibers
Source: Sci Rep. 2024 Oct 13;14:23947. doi: 10.1038/s41598-024-75249-9 (PMC11471788; doi:10.1038/s41598-024-75249-9)
Supplement: Supplementary file 1 — Supplementary Information. [file 41598_2024_75249_MOESM1_ESM.pdf]

Supplementary Information to:

Characterizing Temporal Stability of Supercontinuum Generation in Higher-Order Modes supported by Liquid-Core Fibers

Johannes Hofmann[1], Ramona Scheibinger[1], and Markus A. Schmidt[1,2,*]

[1]Leibniz Institute of Photonic Technology, Albert-Einstein-Str. 9, 07745 Jena, Germany
[2]Otto Schott Institute of Materials Research (OSIM), Friedrich-Schiller-University Jena, Fraunhoferstr. 6, 07743 Jena, Germany
[*]markus-alexander.schmidt@uni-jena.de

Sec. S1: Comparison of first, last and average spectrum

To qualitatively demonstrate the stability of the generated supercontinuum generation (SCG), selected output spectra are compared as follows. Specifically, the first spectrum taken at the beginning of the measurement series (orange line in Fig. S1) is compared with the last spectrum taken after 24 hours (blue line in Fig. S1), along with the average spectrum obtained from all measurements (green line in Fig. S1). As shown on both the logarithmic (dashed lines) and linear (solid lines) scales, (i) the first and last spectra overlap significantly, and (ii) the average spectrum closely matches the two individual spectra. These results clearly demonstrate stable supercontinuum generation throughout the 24-hour measurement period.

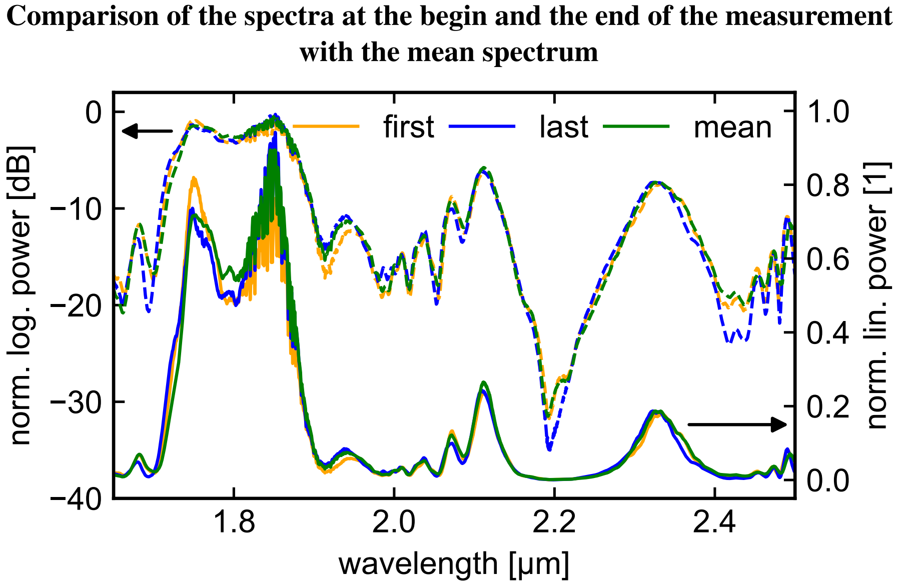

Figure S1. Spectral distribution of three selected output spectra (orange: first spectrum at the beginning of the measurement series, blue: last spectrum taken after 24 hours, green: average spectrum obtained from all spectra considered in this work). The dashed lines refer to the data in dB scale (left axis), the solid curves to the same data in linear scale (right axis).

Sec. S2: Quantifying the stability using a suitable metric

In response to the Reviewer's comment, a statistical analysis was performed to quantify the stability of the supercontinuum generation process at the highest input power used in this study. To ensure meaningful results, the data analysis was focused on the spectral intervals containing the highest spectral powers, as these are typically of most interest. The positions of these intervals relative to the average of all recorded spectra are shown in Fig. 3(f) (green areas).

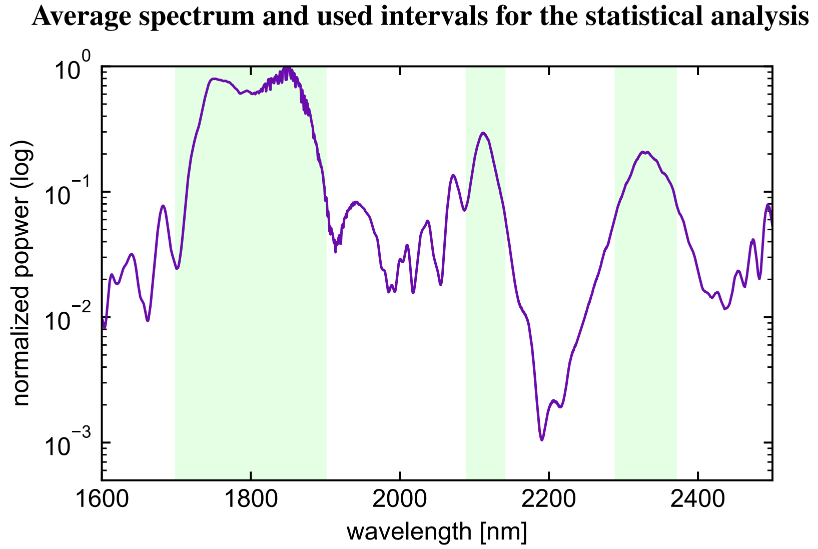

Figure S2. Average output spectrum obtained from all spectra considered in this work. The light green areas indicate the three spectral intervals that contain intense spectral features and thus have been considered in the statistical analysis (same as shown in Fig. 3(f)).

The key parameter characterizing temporal stability is the standard deviation. In the context of this study, this parameter is defined for a given wavelength λ_0 as follows:

$$\overline{P}(\lambda_0) = \frac{1}{N_s}\sum_{i=1}^{N_s} P_i(\lambda_0)$$

$$\sigma_0 = \sqrt{\frac{1}{N_s}\sum_{i=1}^{N_s}\left(P_i(\lambda_0) - \overline{P}(\lambda_0)\right)^2}$$

where N_s is the number of measured spectra, and $P_{\rm i}$ and $\bar{P}$ are the individual and average power values at λ_0 (both in linear scale). The relative spectral deviation RSD, which quantifies the relative variation at a given wavelength, is then defined as

$$RSD(\lambda_0) = \frac{\sigma_0(\lambda_0)}{\overline{P}(\lambda_0)}.$$

To summarize the temporal variations in a single benchmark parameter, the standard deviation was averaged over the spectral interval of interest $\Delta\lambda$. This approach yields the mean standard deviation and allows an overall assessment of the variations within the interval considered, providing a clear indication of stability:

$$\overline{RSD}(\Delta\lambda) = \frac{1}{N_\lambda}\sum_{i=1}^{N_\lambda} RSD(\lambda_i)$$
